# Lotus-Inspired Multiscale Superhydrophobic AA5083 Resisting Surface Contamination and Marine Corrosion Attack

**DOI:** 10.3390/ma12101592

**Published:** 2019-05-15

**Authors:** Binbin Zhang, Weichen Xu, Qingjun Zhu, Shuai Yuan, Yantao Li

**Affiliations:** 1 CAS Key Laboratory of Marine Environmental Corrosion and Bio-fouling, Institute of Oceanology, Chinese Academy of Sciences, No.7 Nanhai Road, Qingdao 266071, China; w.xu@qdio.ac.cn (W.X.); zhuqingjun@qdio.ac.cn (Q.Z.); ys520399@163.com (S.Y.); 2 Open Studio for Marine Corrosion and Protection, Pilot National Laboratory for Marine Science and Technology (Qingdao), No.1 Wenhai Road, Qingdao 266237, China; 3 Center for Ocean Mega-Science, Chinese Academy of Sciences, No.7 Nanhai Road, Qingdao 266071, China; 4 School of Civil Engineering, Qingdao University of Technology, No. 11 Fushun Road, Qingdao 266033, China

**Keywords:** fluorine free, silanization, superhydrophobic, corrosion protection, self-cleaning

## Abstract

The massive and long-term service of 5083 aluminum alloy (AA5083) is restricted by several shortcomings in marine and industrial environments, such as proneness to localized corrosion attack, surface contamination, etc. Herein, we report a facile and cost-effective strategy to transform intrinsic hydrophilicity into water-repellent superhydrophobicity, combining fluorine-free chemisorption of a hydrophobic agent with etching texture. Dual-scale hierarchical structure, surface height relief and surface chemical elements were studied by field emission scanning electron microscopy (FE-SEM), atomic force microscopy (AFM), energy dispersive X-ray spectroscopy (EDS) and X-ray photoelectron spectroscopy (XPS), successively. Detailed investigations of the wetting property, self-cleaning effect, NaCl-particle self-propelling, corrosion and long-term behavior of the consequent superhydrophobic AA5083 surface were carried out, demonstrating extremely low adhesivity and outstanding water-repellent, self-cleaning and corrosion-resisting performance with long-term stability. We believe that the low cost, scalable and fluorine-free transforming of metallic surface wettability into waterproof superhydrophobicity is a possible strategy towards anti-contamination and marine anti-corrosion.

## 1. Introduction

Metals and their alloys are central engineering materials in numerous industrial fields. As a typical representative, 5083 aluminum alloys (AA5083), featuring a high strength-to-weight ratio and good weldability, are widely employed in military equipment, marine constructions, automobile manufacture and aerospace applications. However, proneness to localized corrosion attacks [1,2,3,4] in corrosive environments restricts the large-scale and long-term application of AA5083 materials. Corrosion attacks in aggressive environments can produce a premature failure of AA5083 structural materials, resulting in environmental disruption, enormous economic loss, as well as catastrophic safety accident. Thus, how to endow AA5083 materials with superior corrosion resistance is an extensively concerning issue.

In recent years, inspired from the unique water-repellent property of natural organisms [5,6,7,8,9], the artificial fabrication of bionic superhydrophobic surfaces attracted intensive attention of scientists and engineers owing to their multi-functional applications, such as self-cleaning [10,11], oil–water separation [12,13], drag reduction [14,15], anti-icing/frosting [16,17], microdroplet transportation [18,19], water collection [20,21], marine anti-corrosion [22,23], anti-biofoulings [24,25], etc. It is believed and demonstrated that superhydrophobic surfaces could effectively reduce the solid/liquid interfacial contacts and provide a functional corrosion-resistant barrier. Thus, transforming surface wettability from intrinsic hydrophilicity to water-repellent superhydrophobicity is considered to be a possible strategy resisting marine corrosion attacks [26,27].

At present, much efforts [28,29,30,31] have been devoted to exploring the fabrication and investigating the consequent corrosion-resistant behavior of a superhydrophobic surface on aluminum/aluminum alloy substrates. For instance, Boinovich et al. [32,33] reported a combination of nanosecond laser texturing and fluorinated hydrophobic agent chemisorption, achieving a superhydrophobic aluminum-magnesium alloy with an extremely low corrosion current. Wang et al. [34] used a hydrothermal in situ growth method to fabricate superhydrophobic Mg-Al-layered double hydroxide films on 6061 aluminum alloy substrates, presenting a highly improved corrosion resistance. In our previous reports, we developed an ammonia etching approach [35] and an anodization method [36] followed by 1H,1H,2H,2H-Perfluorodecyltriethoxysilane chemisorption to achieve superhydrophobic surfaces on aluminum/aluminum alloy substrates with multiscale hierarchical topography, and greatly enhanced corrosion inhibition performance.

However, most of the fabrication methods above are hindered by several shortcomings, such as being time-consuming, high-cost and fluorine reagents employment, etc., restricting their large area usage and severely threatening ecological environments and human security. Until now, only limited attempts have been achieved to develop scalable and fluorine-free superhydrophobic aluminum surfaces for marine corrosion protection. So, these issues push us to explore a cost effective, facile and non-fluorinated approach, transforming intrinsically hydrophilic AA5083 with a Wenzel contact [37,38] to superhydrophobic AA5083 with a Cassie–Baxter contact [39,40].

Herein, we report a facile, cost-effective and non-fluorinated fabrication strategy to prepare a superhydrophobic surface on an AA5083 substrate by combining etching texture and a hexadecyltrimethoxysilane (HDTMS) hydrophobic molecules assembly. Detailed studies about the surface morphology and chemical composition were carried out successively. In addition, wetting property, self-cleaning ability, corrosion-resisting behavior and long-term stability were investigated to display the typical characteristics and promising functional applications. 

## 2. Experimental Section

### 2.1. Materials and Reagents

A 5083 aluminum alloy (AA5083) plate with a 0.3 mm thickness was obtained from Dongguan Wanxing Metal Co., Ltd. (Dongguan, China), and tailored into 25 mm × 20 mm specimens. The main composition of the pristine AA5083 substrate was 4.0–4.9% Mg, 0.4–1.0% Mn, 0.25% Zn, 0.4% Si, 0.15% Ti, 0.1% Cu, 0.05–0.25% Cr, 0.1–0.4% Fe and the balance was Al. Sodium hydroxide (NaOH), sodium chloride (NaCl), methylene blue trihydrate (C_16_H_18_ClN_3_S·3H_2_O), ethanol absolute and graphite powder were purchased from Sinopharm Chemical Reagent Co., Ltd. (Beijing, China). Manganese monoxide (MnO) was bought from Aladdin Industrial Corporation. Hexadecyltrimethoxysilane (C_19_H_42_O_3_Si, HDTMS) was received from J&K Scientific Ltd. (Shanghai, China). All experimental reagents mentioned above were used as received without further purification.

### 2.2. Preparation of Superhydrophobic AA5083

**Figure 1:**
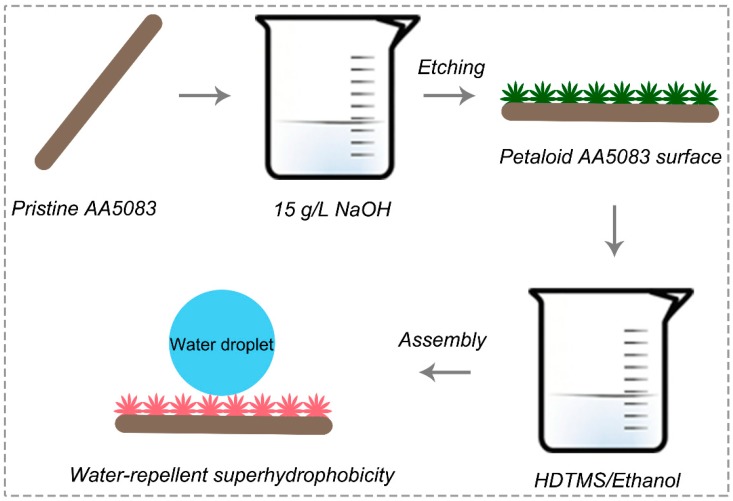
Schematic illustration of the fabrication process.

The brief schematic illustration of the fabrication process is presented in Figure 1, involving an etching-texture process and HDTMS assembly. Firstly, the pristine AA5083 substrates were sanded through SiC sandpaper with different grades (400, 800, 1200, etc.) and cleaned by sonication in ethanol and deionized water for more than 5 min, respectively. The cleaned and pristine AA5083 specimens were rinsed with deionized water and dried under an air blower before etching. Subsequently, the pristine AA5083 samples were immersed in 15 g/L sodium hydroxide aqueous solution for 30 min to accomplish the etching texture. After deionized water cleaning, ethanol cleaning and an 80 °C oven drying treatment, the etching-textured AA5083 specimens were immersed in a 3 vol.% HDTMS/ethanol solution for 1 h to chemically assemble HDTMS molecules. After the modification process, the modified AA5083 specimens were heated at 120 °C in a drying oven for 20 min.

### 2.3. Characterization

The surface topography of pristine and as-fabricated superhydrophobic AA5083 surfaces were characterized by field emission scanning electron microscopy (FE-SEM, FEI Nova Nano SEM450, Hillsboro, OR, USA) and atomic force microscopy (AFM, Bruker Multimode 8, Karlsruhe, Germany). The AFM images were obtained under tapping mode. Energy dispersive X-ray spectroscopy (EDS, Oxford X-Max^N^50, Hillsboro, OR, USA) and X-ray photoelectron spectroscopy (XPS, Thermo Scientific Escalab 250Xi, Massachusetts, MA, USA) were performed to determine the existence of the key elements upon the specimens. The XPS measurements were performed using a monochromated Al Kα irradiation and the chamber pressure was 3 × 10^−8^ Torr during the test. The binding energy of adventitious carbon C1s (284.8 eV) was used as a basic reference. A Dataphysics OCA25 instrument (Stuttgart, Germany) was used to measure the static water contact angles and sliding angles of the as-prepared superhydrophobic AA5083 samples. For each measurement, at least three different positions were performed to obtain average data.

### 2.4. Electrochemical Test

The electrochemical tests were all carried out in a 3.5 wt.% NaCl aqueous solution through an Ametek Parstat 4000+ electrochemical workstation. A typical three-electrode measure system, including counter electrode (Pt sheet), reference electrode (saturated silver/silver chloride) and working electrode (pristine/superhydrophobic AA5083), was employed to proceed with the electrochemical tests. Prior to the test, the working electrode was exposed to a 3.5 wt.% NaCl aqueous solution for more than 1 h, achieving a stable measuring system. Electrochemical impedance spectroscopy (EIS) was measured under OCP (open circuit potential) at a frequency range of 100 kHz–10 MHz. *ZsimpWin* software was utilized to analyze and fit the EIS data for anti-corrosion evaluation. 

## 3. Results and Discussion

### 3.1. Surface Morphology and Wettability Behavior

**Figure 2:**
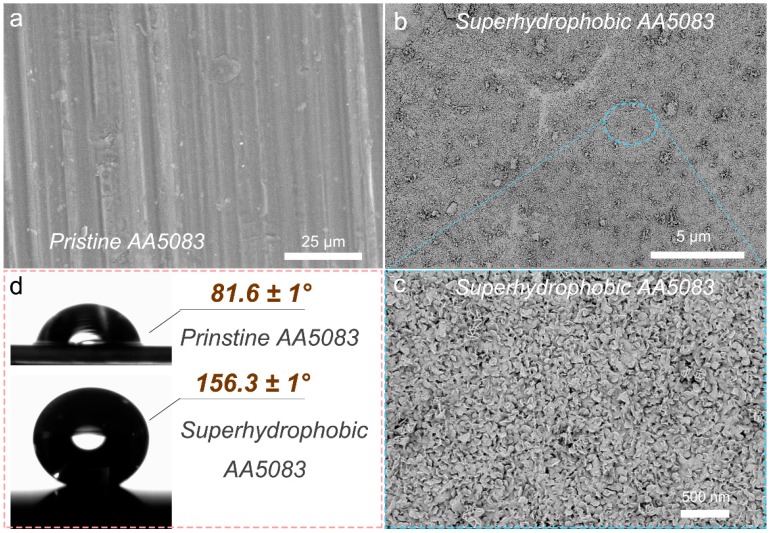
FE-SEM images (**a**–**c**) and static contact angles (**d**) of pristine 5083 aluminum alloy (AA5083) and as-fabricated superhydrophobic AA5083 surfaces.

The surface topography of pristine AA5083 and superhydrophobic AA5083 surfaces were characterized by FE-SEM. Figure 2 displays the SEM images of the pristine AA5083 and superhydrophobic AA5083 surface. As for pristine AA5083 shown in Figure 2a, some micro-grooves/scratches can be seen, which is attributed to the pre-treatment of the pristine AA5083 specimen. The surface of the pristine AA5083 is relatively smooth. For the etching-textured superhydrophobic AA5083 surface, displayed in Figure 2b, some micro-textured rough structure can be found. Figure 2c shows the higher magnification image of Figure 2b and presents an obviously nano-scale petaloid surface architecture of the as-prepared superhydrophobic AA5083 sample. These micro-nano hierarchical structures contribute to the increase of surface roughness and provide sufficient structural clearance for the formation of the trapped air cushion between the solid surface and the water droplet, which is beneficial for the final Cassie–Baxter contact state. Figure 2d shows the static water contact angle of pristine AA5083 and superhydrophobic AA5083 surface. The contact angle of the pristine AA5083 surface is about 81.6° ± 1°. After the etching texture and HDTMS assembly, however, the contact angle of the as-prepared superhydrophobic AA5083 surface is about 156.3° ± 1° with a sliding angle lower than 1°. The etching-textured, dual-scale surface rough structure and the low surface energy of the HDTMS molecules endowed the AA5083 substrate with a high static water contact angle and a low sliding angle.

**Figure 3:**
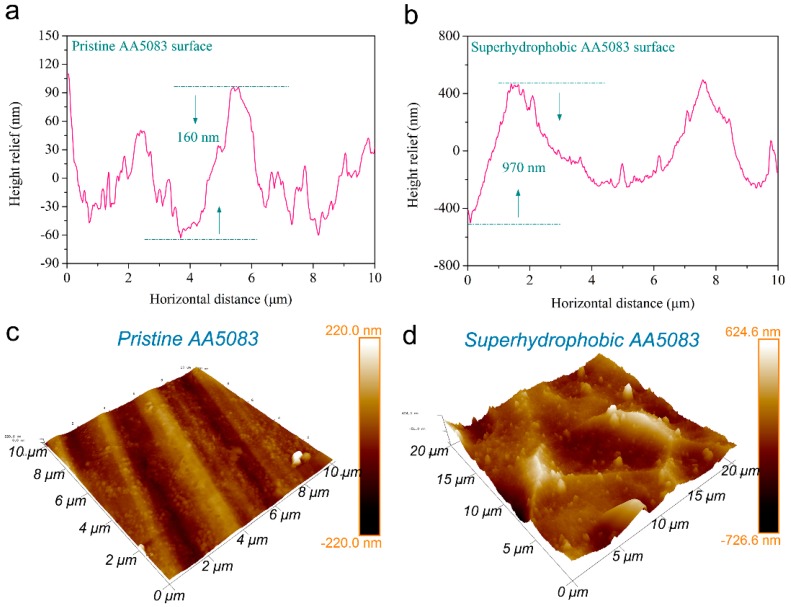
Surface relief and 3D atomic force microscopy (AFM) images of pristine AA5083 (**a**,**c**) and superhydrophobic AA5083 (**b**,**d**).

As is well known, wetting behavior of a solid surface is mainly determined by roughness and surface energy. The microscale roughness and height relief of the pristine and as-fabricated superhydrophobic AA5083 surfaces were revealed by AFM, as shown in Figure 3. Figure 3a,b display the topographic fluctuation of pristine AA5083 and superhydrophobic AA5083 specimens. It was found that the height relief of the pristine AA5083 substrate was about 160 nm. On the contrary, the height relief of the as-prepared superhydrophobic AA5083 was approximately 970 nm, presenting a significant improvement. Figure 3c,d show the 3D AFM images of pristine AA5083 and superhydrophobic AA5083 surfaces. The surface roughness can be clearly observed and contrasted. Generally, *R*_a_ (average roughness), *R*_q_ (root mean square roughness) and *R*_max_ (maximum roughness) were utilized to present surface roughness. In this case, the surface of the pristine AA5083 was relatively smooth with *R*_a_, *R*_q_ and *R*_max_ values (scanning area 10 μm × 10 μm) being 33.3 nm, 42.8 nm and 444 nm, respectively. While for the as-prepared superhydrophobic AA5083 surface, the *R*_a_, *R*_q_ and *R*_max_ values (scanning area 20 μm × 20 μm) were apparently increased to 107 nm, 142 nm and 1229 nm, respectively. As discussed above, the resultant superhydrophobic AA5083 surface features an obviously improved surface roughness. The etching-textured process contributes to this roughness enhancement, which is in favor of the air cushion formation and Cassie–Baxter contact of the water/solid/air interface.

### 3.2. Low Surface Adhesivity

**Figure 4:**
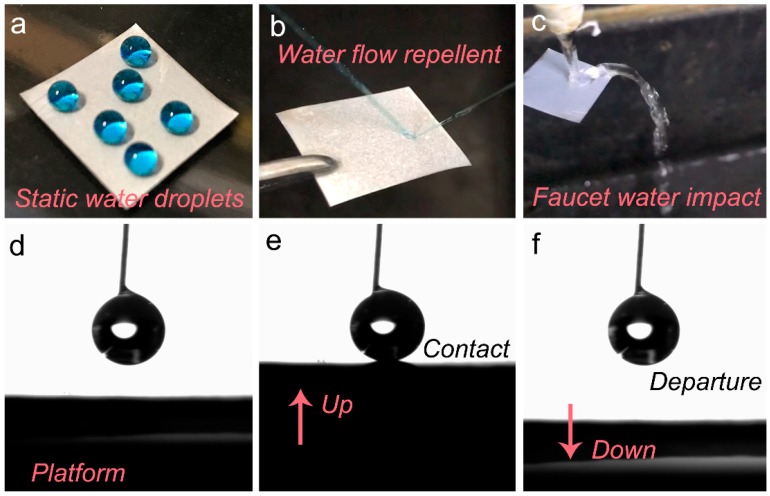
Optical images of the as-prepared superhydrophobic AA5083 surface (**a**) with spherical water droplets, (**b**) with a jet of water flow, (**c**) with faucet water impact, and (**d**–**f**) with platform movement to contact and departing of the water droplet.

The larger etching-textured roughness and lower HDTMS surface energy played key roles in the resultant water-repellent superhydrophobicity. Figure 4a displays the optical image of spherical water droplets, illustrating a waterproof property. Figure 4b,c show the surface response with a dynamic jet of water flow and faucet water impact. It can be clearly seen that the water flow jet and faucet water cannot remain on the superhydrophobic AA5083 surface. The water flow reflects, bounces and finally reflecting/rolling away easily from the specimen. Figure 4d–f presents the optical images of platform movement to contact and departing of the water droplet using the Dataphysics OCA25 instrument. With the gradually approaching, contacting and departing, the as-prepared superhydrophobic AA5083 surface could completely depart from the water droplet after tight contact, suggesting an extremely low adhesivity.

The above wetting property and low adhesivity of the as-prepared superhydrophobic AA5083 can be explained by the combination action of the two-tier hierarchical rough structure and the HDTMS modification. Hierarchical petaloid rough structures benefit from the formation of the trapped air cushion, which significantly restrain the interfacial contact of the water–solid phase. Furthermore, the introduction of HDTMS molecules decrease the surface energy and further suppresses the penetration of water droplets into the surface structure. Thus, it can be concluded from the interaction between water and the superhydrophobic AA5083 surface that the water-repellent superhydrophobicity is attributed to the microscale roughness of the etching-textured surface and the low surface energy of HDTMS molecules. 

### 3.3. Chemical Composition

**Figure 5:**
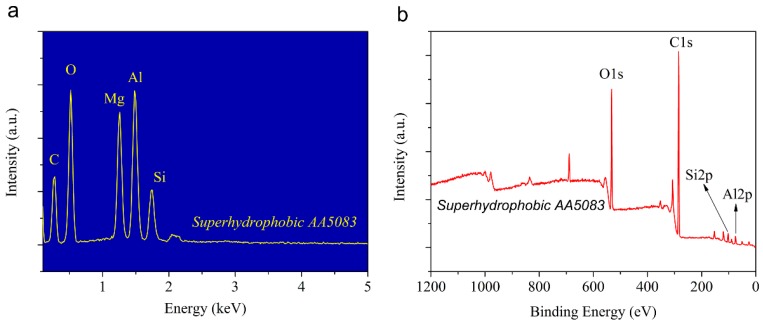
Energy dispersive X-ray spectroscopy (EDS) and X-ray photoelectron spectroscopy (XPS) spectra of the fabricated superhydrophobic AA5083 sample. (**a**) EDS spectrum, and (**b**) XPS spectrum.

The chemical composition of the pristine and HDTMS assembly superhydrophobic AA5083 surfaces were characterized by EDS (Figure 5a) and XPS (Figure 5b). The surface is rich in C, O, Mg, Al and Si elements as evidenced by the EDS spectrum, preliminarily verifying the assembly of the long carbon-chain tail on the as-prepared superhydrophobic AA5083 surface. As is well known, the XPS spectra present a surface chemical composition with a detecting depth of a few nanometers. The detailed elemental composition was further investigated through an XPS spectrum, as shown in Figure 5b. It is obvious that C, O, Si and Al elements were detected and demonstrated in the XPS spectrum of the as-prepared superhydrophobic AA5083 surface. Strong binding energy located at 284.7 eV, 532.4 eV, 102.3 eV and 74.6 eV were confirmed and ascribed to C 1s, O 1s, Si 2p and Al 2p, respectively, further confirming the existence of HDTMS species in the superhydrophobic AA5083 substrate. The C 1s, O 1s and Si 2p peaks in XPS spectra of the as-prepared superhydrophobic AA5083 sample were contributed to the HDTMS molecule assembly. This is in accordance with the EDS spectrum. It can be concluded, based on the analyses of surface topography, microscale roughness, wetting property and chemical composition, that the AA5083 substrates were endowed with excellent water repellent superhydrophobicity.

### 3.4. Self-Cleaning Ability and NaCl Self-Propelling

**Figure 6:**
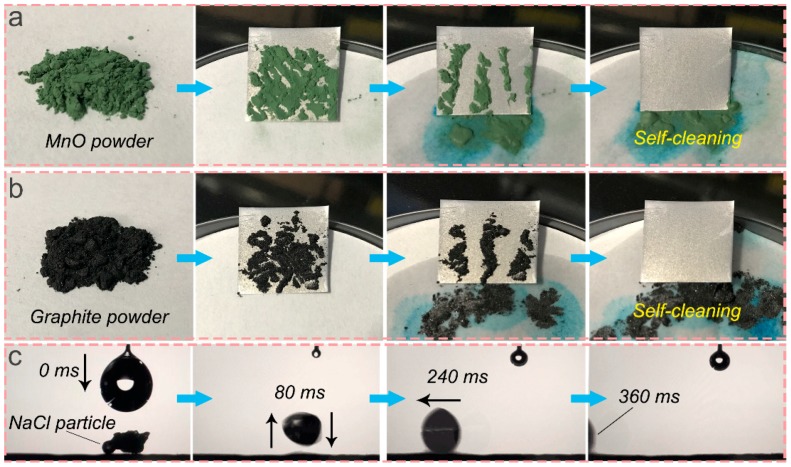
Self-cleaning ability with different surface contaminations: (**a**) MnO powder, (**b**) graphite powder, and (**c**) NaCl self-propelling property.

As for real-world applications, the self-cleaning ability of superhydrophobic material is an essential and promising function. In this work, MnO powder and graphite powder were successively applied as surface contaminations of the as-prepared superhydrophobic AA5083 samples. The superhydrophobic AA5083 specimen was firstly inclined at an angle lower than 10°. Water droplets were subsequently dropped from above to evaluate the self-cleaning effect of the specimen. Figure 6a,b display the optical photos of the self-cleaning process. Given the excellent water repellence of the superhydrophobic AA5083 specimen, the water droplets can instantaneously roll off the sample surface, effectively picking up and taking away the MnO powder and graphite powder without difficulty. The water droplets rolled down the superhydrophobic AA5083 sample without moistening the solid surface, leaving several traces of rolling upon the surface. After washing, the contaminated AA5083 samples were totally clean and had no differences compared to the original uncontaminated specimen.

Figure 6c presents the NaCl self-propelling property of the superhydrophobic AA5083 surface. The NaCl particle was put on the surface of the sample. After the water droplet fell to the surface, the NaCl particle was fused with the water droplet. The outstanding water-repellence of the superhydrophobic AA5083 surface overcame the gravity of the NaCl particle, exhibiting a typical droplet/NaCl bouncing and deformation. An extremely low sliding angle propelled the droplet/NaCl coalition.The NaCl self-propelling process only takes 360 ms from the water droplet falling to the final sliding away. The self-cleaning and NaCl self-propelling ability mentioned above can be mainly ascribed to the extremely low surface energy, low adhesivity and water-repellence property of the surface. Therefore, it can be concluded that the fabricated superhydrophobic AA5083 substrate possesses an excellent self-cleaning ability resisting different surface contaminations.

### 3.5. Marine Corrosion Protection

**Figure 7:**
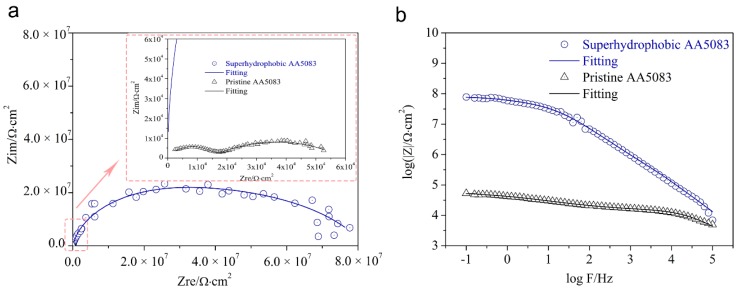
(**a**) Electrochemical impedance spectroscopy (EIS) plots, and (**b**) Bode plots of log |Z| vs. frequency and fittings of the pristine AA5083 and superhydrophobic AA5083 surfaces.

In order to quantitatively characterize the corrosion behavior of the as-prepared superhydrophobic AA5083 film, we carried out and analyzed the electrochemical impedance spectroscopy (EIS) in an open circuit condition of a 3.5 wt.% NaCl aqueous corrosive solution. Figure 7a presents the EIS plots and fittings of pristine AA5083 and superhydrophobic AA5083 substrates. It can be seen from superhydrophobic AA5083 EIS plots that the diameter of the capacitive loop is much larger than that of the pristine AA5083 specimen. Figure 7b displays Bode plots of log |Z| vs. frequency and fittings of the pristine AA5083 and superhydrophobic AA5083 surfaces, presenting a three orders of magnitude higher impedance modulus value than that of the pristine AA5083 substrate. The anti-wetting and water-repellent property of the as-fabricated superhydrophobic AA5083 surface contributes to the remarkable improvement of impedance modulus.

**Figure 8:**
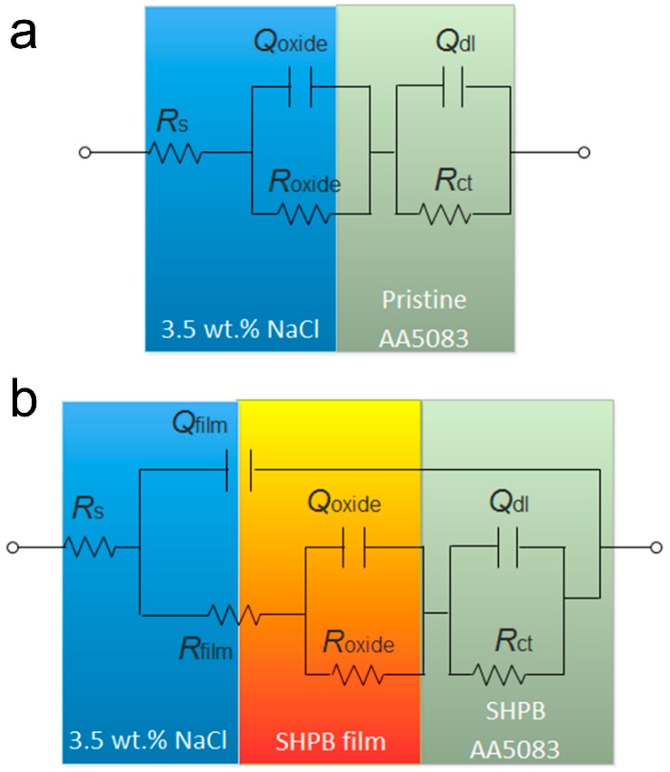
Equivalent circuit of (**a**) pristine AA5083 and (**b**) superhydrophobic AA5083.

Different equivalent circuits were employed to analyze the EIS results through *ZsimpWin* software, as shown in Figure 8. Figure 8a,b display the equivalent circuit of pristine AA5083 and superhydrophobic AA5083 specimens. For the convenience of EIS data analyzing, *R*_s_, *R*_film_, *R*_oxide_ and *R*_ct_ in Figure 8 represent solution resistance, superhydrophobic film resistance, oxide resistance and charge transfer resistance, respectively. *Q*_film_, *Q*_oxide_ and *Q*_dl_ represent the constant phase elements (CPE) modelling capacitance of the as-prepared superhydrophobic film, the oxide layer and the double-layer, respectively. Wherein, the impedance of the CPE could be defined as 1/*Y*_0_(*jω*)^n^ [41,42], where *Y*_0_, *j*, *ω* and n represent the modulus, imaginary number, angular frequency and the phase, respectively.

The fitted electrochemical parameters are shown in Table 1. In General, *R*_ct_ is utilized to calculate and evaluate the corrosion resisting property of the fabricated protective layers. From Table 1, it can be clearly seen that the *R*_ct_ of the as-prepared superhydrophobic AA5083 specimen is 1.14 × 10^6^ Ω·cm^2^, while the *R*_ct_ of the pristine AA5083 sample is only 4.44 × 10^4^ Ω·cm^2^. The *R*_ct_ of superhydrophobic AA5083 is two orders of magnitude higher than that of pristine AA5083, demonstrating a remarkable enhanced corrosion-resisting performance. In addition, the *Q*_dl_ of pristine AA5083 and as-prepared superhydrophobic AA5083 was 6.77 × 10^−6^ Ω^−1^·cm^−2^·s^n^ and 3.54 × 10^−10^ Ω^−1^·cm^−2^·s^n^, respectively. The lower *Q*_dl_ value and higher *R*_ct_ value of the as-prepared superhydrophobic AA5083 substrate suggest that the charge transfer process of corrosive ions occurs with difficulty.

In general, the *R*_ct_ value is utilized to calculate the inhibition efficiency (*η*) of the protective film using the formula *η* = (*R*_ct_ − *R*_ct_^0^)/*R*_ct_ [43], in which *R*_ct_ and *R*_ct_^0^ represent the charge transfer resistance of the superhydrophobic AA5083 specimen and the pristine AA5083 specimen. It was calculated using the *R*_ct_ and *R*_ct_^0^ values discussed above that the inhibition efficiency in this case was approximately 96.11%, indicating an impressive performance resisting marine corrosion attack.

**Figure 9:**
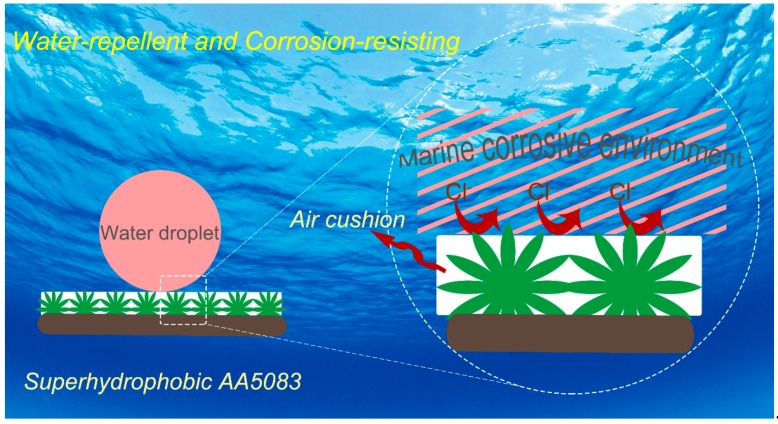
Anticorrosion mechanism of the as-prepared superhydrophobic AA5083.

Figure 9 shows the anticorrosion mechanism of the as-fabricated superhydrophobic AA5083 surface. When the pristine AA5083 is exposed to environments containing aggressive corrosive ions, the natural oxide film breaks down at specific points leading to the formation of localized corrosion on AA5083 surface, viz. pitting corrosion or intergranular corrosion. On the contrary, the as-prepared superhydrophobic AA5083 surface could trap air within the micro-nano petaloid hierarchical structure, presenting a greatly decreasing fraction of the water/solid interface. The superior corrosion resistance is mainly attributed to the air cushion trapped in the petaloid rough structure, which suppresses the penetration and diffusion of aggressive corrosion species crossing the superhydrophobic protective film to the underlying AA5083 substrate. So, it can be implied that the as-fabricated superhydrophobic AA5083 specimen could largely improve the corrosion resistance of the substrate, displaying an outstanding protection ability towards marine corrosion attack.

### 3.6. Long-Term Stability

**Figure 10:**
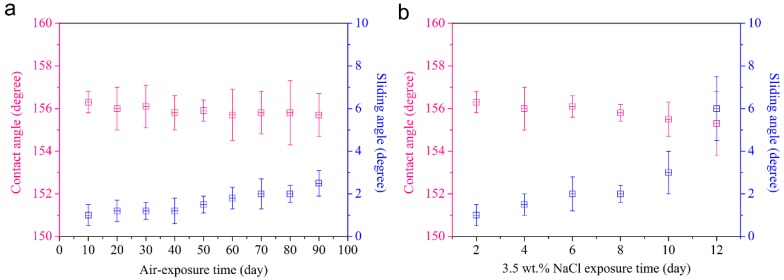
The variation of the contact angle and sliding angle under different exposure time in (**a**) air and (**b**) 3.5 wt.% NaCl aqueous solution.

For practical applications, it is significant to characterize the stability of the superhydrophobic AA5083 specimen subjected to air exposure and a corrosive solution immersion for prolonged period. In this case, we carried out air exposure and a 3.5 wt.% NaCl aqueous solution immersion experiments to evaluate the durability of the as-produced superhydrophobic AA5083 substrate, as shown in Figure 10a,b. After 90 days of air exposure and 12 days of a 3.5 wt.% NaCl immersion/contact of the as-fabricated superhydrophobic AA5083 surface, it can be found from the contact angle and sliding angle variation that the as-prepared surface causes nearly no degradation of water-repellent superhydrophobicity with a contact angle higher than 155°, indicating outstanding long-term stability. The multiscale hierarchical structure and chemisorbed HDTMS hydrophobic molecules facilitate the formation of an air cushion, significantly contributing to the eventual water-repellence, air-exposure stability and corrosive-medium immersion stability.

## 4. Conclusions

In conclusion, to successfully design a lotus-inspired superhydrophobic AA5083 surface with a facile, cost-effective and fluorine-free strategy, we provide an approach combining etching texture followed by chemisorption of HDTMS hydrophobic molecules. It was shown that the surface topography of the as-prepared superhydrophobic AA5083 possesses a micro-nano hierarchical petaloid structure with a contact angle of 156.3° ± 1° and a sliding angle lower than 1°. The wetting behavior investigations presented water-repellence, extremely low adhesivity and a self-cleaning ability. The EIS and fitting results showed that the *R*_ct_ of the as-prepared superhydrophobic AA5083 specimen is two orders of magnitude higher than that of the pristine AA5083. In addition, the *Q*_dl_ of the as-prepared superhydrophobic AA5083 was four orders of magnitude lower than the pristine AA5083. The inhibition efficiency in this case was approximately 96.11%. In addition, after 90 days of air exposure and 12 days of a 3.5 wt.% NaCl immersion/contact, the as-fabricated superhydrophobic AA5083 surface sustained a durable and stable superhydrophobicity. Therefore, the resultant superhydrophobic AA5083 surface possesses superior corrosion-resisting performance and long-term stability. We greatly anticipate that this research work has important significance for the large-scale manufacturing of water-repellent superhydrophobic surfaces for long-term marine and industrial applications.

## Figures and Tables

**Table 1:** The electrochemical parameters of simulated pristine AA5083 and superhydrophobic AA5083 surfaces in 3.5 wt.% NaCl aqueous solution.

Parameters	Specimens	
	Pristine AA5083	Superhydrophobic AA5083
*R*_s_ (Ω·cm^2^)	2.43	4.09
*Q*_film_ (Ω^−1^·cm^−2^·s^n^)	/	1.19 × 10^−10^
*n*_1_	/	1
*R*_film_ (Ω·cm^2^)	/	8.24 × 10^7^
*Q*_oxide_ (Ω^−1^·cm^−2^·s^n^)	5.84 × 10^−9^	1.66 × 10^−9^
*n*_2_	0.79	0.59
*R*_oxide_ (Ω·cm^2^)	1.47 × 10^4^	8.29 × 10^7^
*Q*_dl_ (Ω^−1^·cm^−2^·s^n^)	6.77 × 10^−6^	3.54 × 10^−10^
*n*_3_	0.44	0.80
*R*_ct_ (Ω·cm^2^)	4.44 × 10^4^	1.14 × 10^6^
*η* (%)	/	96.11
